# Construction and validation of a transcription factors-based prognostic signature for ovarian cancer

**DOI:** 10.1186/s13048-021-00938-2

**Published:** 2022-02-28

**Authors:** Qingyuan Cheng, Liman Li, Mingxia Yu

**Affiliations:** 1 Department of Andrology/Sichuan Human Sperm Bank, West China Second University Hospital, Sichuan University, Chengdu, Sichuan P. R. China; 2 Key Laboratory of Birth Defects and Related Diseases of Women and Children of Ministry of Education, West China Second University Hospital, Sichuan University, Chengdu, China; 3 Department of Clinical Laboratory, Zhongnan Hospital of Wuhan University, Wuhan, China

**Keywords:** ovarian cancer, transcription factor, signature, prognosis, nomogram

## Abstract

**Background:**

Ovarian cancer (OC) is one of the most common and lethal malignant tumors worldwide and the prognosis of OC remains unsatisfactory. Transcription factors (TFs) are demonstrated to be associated with the clinical outcome of many types of cancers, yet their roles in the prognostic prediction and gene regulatory network in patients with OC need to be further investigated.

**Methods:**

TFs from GEO datasets were collected and analyzed. Differential expression analysis, WGCNA and Cox-LASSO regression model were used to identify the hub-TFs and a prognostic signature based on these TFs was constructed and validated. Moreover, tumor-infiltrating immune cells were analyzed, and a nomogram containing age, histology, FIGO_stage and TFs-based signature were established. Potential biological functions, pathways and the gene regulatory network of TFs in signature was also explored.

**Results:**

In this study, 6 TFs significantly associated with the prognosis of OC were identified. These TFs were used to build up a TFs-based signature for predicting the survival of patients with OC. Patients with OC in training and testing datasets were divided into high-risk and low-risk groups, according to the median value of risk scores determined by the signature. The two groups were further used to validate the performance of the signature, and the results showed the TFs-based signature had effective prediction ability. Immune infiltrating analysis was conducted and abundance of B cells naïve, T cells CD4 memory resting, Macrophages M2 and Mast cells activated were significantly higher in high-risk group. A nomogram based on the signature was established and illustrated good predictive efficiencies for 1, 2, and 3-year overall survival. Furthermore, the construction of the TFs-target gene regulatory network revealed the potential mechanisms of TFs in OC.

**Conclusions:**

To our best knowledge, it is for the first time to develop a prognostic signature based on TFs in OC. The TFs-based signature is proven to be effective in predicting the survival of patients with OC. Our study may facilitate the clinical decision-making for patients with OC and help to elucidate the underlying mechanism of TFs in OC.

**Supplementary Information:**

The online version contains supplementary material available at 10.1186/s13048-021-00938-2.

## Background

Ovarian cancer (OC) is one of the most common and lethal malignant tumors, threatening global female health [1]. Despite advances in diagnosis and treatment of OC during the past decades, the clinical outcome of patients with OC remains dismal. OC has the highest mortality rate of all gynecological tumors, and the 5-year survival of patients with OC was less than 50% [2]. Although multiple genetic alterations are implicated in the pathogenesis and development of OC, the exact molecular mechanism and regulatory network behind this highly complex cancer remains unclear [3]. Hence, it is imperative to construct novel prognostic signature and investigate the underlying molecular regulation of OC in order to improve prognostic prediction and treatment options of this malignant tumor.

Transcription factors (TFs) are DNA-binding proteins that play essential roles in controlling genes expression [4]. Many TFs have been demonstrated to be critical for regulating the tumorigenesis and progression of multiple cancers, such as pro-inflammatory TFs and hypoxia-inducible factors [5]. Previous studies have found that epithelial-mesenchymal transition inducing-TFs were strongly associated with the prognosis of head and neck squamous cell carcinoma [6]. E2F transcription factors were also identified as new biomarkers for the prognosis of breast cancer [7]. The potential of TFs in carcinogenesis, development and prognosis of cancers deserves more in-depth research.

Due to the urgent demand for a reliable, prompt and accurate prognostic stratification to help clinical decision-making of cancers, many risk score models were identified for predicting the clinical outcome of patients with OC. However, those signatures were mostly focus on genes, miRNAs or lncRNAs [8, 9]. TFs, functioning as crucial factors in progression of OC, were less studied [10]. Guo et al. constructed a stage-specific TF-lncRNA regulatory networks and further identified a TF-associated lncRNAs-based risk score model that could be useful for prognosis stratification and the identification of therapeutic targets in OC [11, 12]. Above-mentioned findings ascertained the prognostic value of TFs in OC and the potential of TFs for establishing an effective signature for predicting survival of patients with OC.

**Scheme 1:**
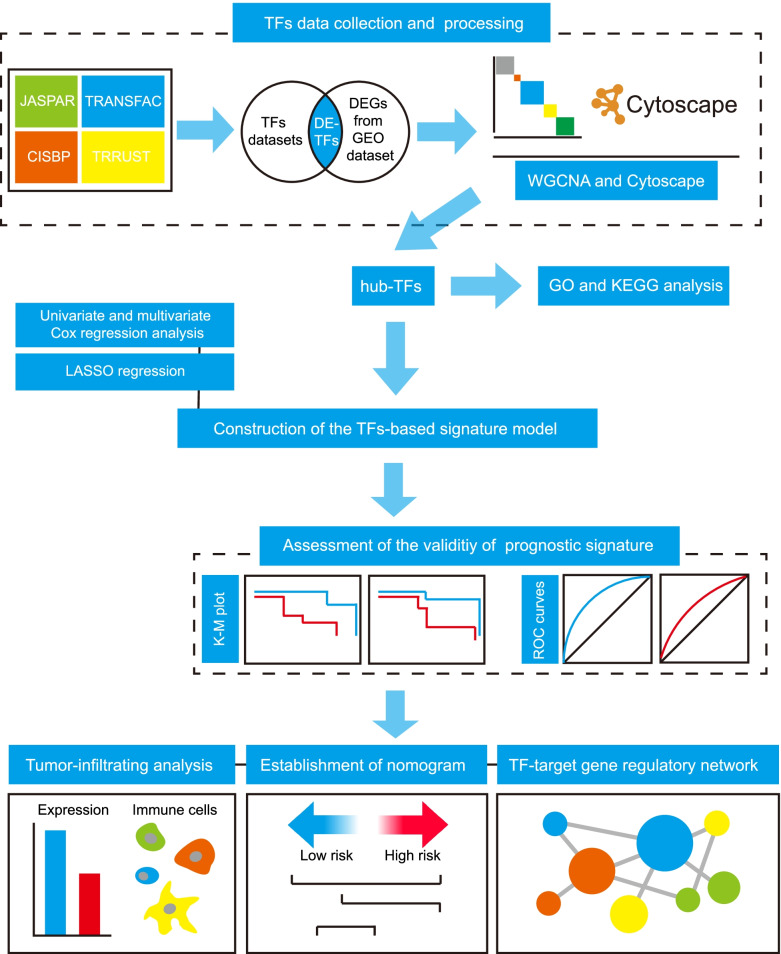
The flow diagram of this study

In the present study, we aimed to construct a prognostic signature for predicting the survival of patients with OC using TFs data mainly from GEO database **(**Scheme 1**)**. Firstly, differentially expressed TFs (DE-TFs) was obtained through the intersection of the DEGs and several TFs databases. Then DE-TFs co-expression network was constructed using WGCNA, and hub-TFs were identified in significantly OC-related modules by Cytoscape. Cox-LASSO regression was performed to finally generate a TFs-based signature containing 6 TFs. According to the risk scores in the signature, patients with OC were divided into high-risk group and low-risk group. The validity of the TFs-based signature was assessed between two groups both in training and testing datasets. The TFs-based signature was further combined with several clinicopathological factors to establish a nomogram that could effectively predict survival probability. In addition, the relative abundance of 22 types tumor infiltrating immune cells in two groups were analyzed using CIBERSORT. Moreover, a TFs-gene target regulatory network was constructed by TRRUST to give insight into the underlying mechanism of the TFs in OC. To our best knowledge, this is the first risk score model based on TFs to predict the survival of patients with OC. The TFs-based signature and nomogram we constructed could be effective in prognosis prediction and have potential to assist clinical decision-making. Our study also identified the hub-TFs and shed lights on the roles of TFs and related gene regulatory network in the occurrence and progression of OC.

## Materials and Methods

### TFs data collection and processing

TFs in 4 commonly used TFs databases in bioinformatic analysis, JASPAR (http://jaspar.genereg.net/), TRANSFAC (http://gene-regulation.com/pub/databases.html), CISBP (http://cisbp.ccbr.utoronto.ca/, including the public data in TRANSFAC) and TRRUST (https://www.grnpedia.org/trrust/) were collected and processed, and the duplicate TFs in each database were removed. The combined TFs database was used for extracting the differentially expressed TFs from differential gene expression profiles of OC.

### Selection of differentially expressed TFs (DE-TFs)

The microarray data of OC in the GSE26712 and GSE140082 datasets were downloaded from GEO database (Table 1, Table S1). We also collected datasets related to OC from the GEO database as candidates for instance GSE81873 and GSE32062. However, these datasets were eventually removed due to lack of adequate data or incomplete survival information. Robust Multichip Average (RMA) in the oligo package in R was performed for background adjustment and normalization of raw CEL files, and normalized data were downloaded directly. At first, differentially expressed genes (DEGs) between tumor tissues and normal tissues in the dataset were analyzed and mined using the empirical *Bayes* method described in the limma package in R. Then, all probe sets were annotated with their corresponding official gene symbols using annotation files downloaded from GEO databases. The mean value of probe expressions data of the same gene was used as the representative value. Next, the total DEGs of OC were determined using the *|logFC| > 0.05* and *P < 0.05* as cut-off criterion. Finally, the intersection of the DEGs and above-mentioned combined TFs database containing 784 TFs were obtained and defined as differentially expressed TFs (DE-TFs).

**Table 1:** The GEO datasets including in this study

Database	GSE26712	GSE140082
Normal	10	0
Cancer	185	380

### Construction of WGCNA and identification of hub-TFs

Weighted Correlation Network Analysis (WGCNA) is a systems biology method that could identify the modules of highly correlated genes and find candidate biomarker genes as well as potential therapeutic targets, according to the links between gene sets and phenotypes. In this study, a co-expression network was constructed using WGCNA package in R based on the DE-TFs. The optimistic soft-thresholding power was screened in order to obtain a TFs co-expression network in accord with scale-free network model. Topological overlap matrix (TOM) was constructed and the hierarchical clustering tree was built based on the dissimilarity matrix (1-TOM) and the dynamic tree cut was used for identifying modules that were significantly associated with the tumorigenesis and development of the OC.

The TFs expression profiles data in the significantly OC-related modules were then analyzed by Cytoscape and the connectivity of each TF was measured using CytoHubba, an add-in in Cytoscape. The TFs in the network were ranked by node degrees as hub-TFs and selected for further analysis.

### Functional enrichment and pathway analysis

To explore the functions and signaling pathways of TFs in the modules that was significantly related with OC screened from WGCNA, Gene Ontology (GO) functional enrichment analysis and Kyoto Encyclopedia of Genes and Genomes (KEGG) pathway analysis were performed using clusterProfiler package in R.

### Construction of the TFs-based signature model

GSE26712 was used as the training set to generate the model in this work. Firstly, univariate Cox regression analysis was performed to identify hub-TFs that was significantly associated with patient outcomes (*P < 0.05*). Then the least absolute shrinkage and selection operator (LASSO*)* regression model was carried out to eliminate the redundant factors and to find the most significant survival-associated TFs. Finally, stepwise multivariate Cox proportional hazards model was employed to construct an optimized risk score model for predicting the clinical outcome of the patients with OC, which was defined as the TFs-based signature. The risk score was calculated according to following formula: 

$$h(t)={h}_0(t)\mathit{\exp}\left({\sum}_{i=1}^n{\beta}_i{K}_i\right)$$

where h_0_(t), n, β_i_ and K_i_ represent the baseline hazard function, number of most significant survival-associated TFs, the coefficient, and level of gene expression, respectively.

### Assessment of the validity of prognostic signature

In order to assess the validity of the TFs-based signature, GSE26712 was used as training dataset. According to the median value of risk score, patients in GSE26712 were divided into two groups (high-risk group and low-risk group) and clinical outcome of patients between two groups were compared by plotting Kaplan-Merier survival curves using survival package in R. Then, GSE140082 was used as testing dataset to further evaluate the validity of the TFs-based signature. The TFs-based signature was also verified by plotting receiver operating characteristic (ROC) curve using survivalROC package in R. Moreover, a heatmap were depicted by clustering TFs expression, risk groups and clinical status of the patients to exhibit performance of the TFs-based signature.

### Analysis of tumor-infiltrating immune cells

To explore the associations between tumor immune infiltration and the risk groups determined by the TFs-based signature, the CIBERSORT algorithm was introduced to infer the relative abundance of 22 types of tumor-infiltrating immune cells using microarray data of patients with OC (https://cibersort.stanford.edu). The profiles of 22 tumor-infiltrating immune cells in the high-risk group and low-risk group were depicted and wilcox test was used to calculate the statistical significance between the two groups.

### Establishment of nomogram

For visualizing the prognostic prediction of patients with OC, a nomogram was generated according to several clinicopathological factors (age, histology, FIGO_stage, Table S1) and the TFs-based signature using rms package in R. The performance of the nomogram was evaluated by calculating Harrell’s concordance index (C-index) and the calibration curves for predicting 1, 2, and 3-year overall survival, which could assess the agreement between the actual observed rates and the predicted survival probability.

### Construction of TF-target gene regulatory network

To explore potential functions of TFs in the prognostic signature and identify genes they may target, a TFs-target gene regulatory network was constructed utilizing TRRUST database and was visualized by Cytoscape. (https://cytoscape.org).

## Results

### Identification of differentially expressed TFs (DE-TFs)

**Fig. 1:**
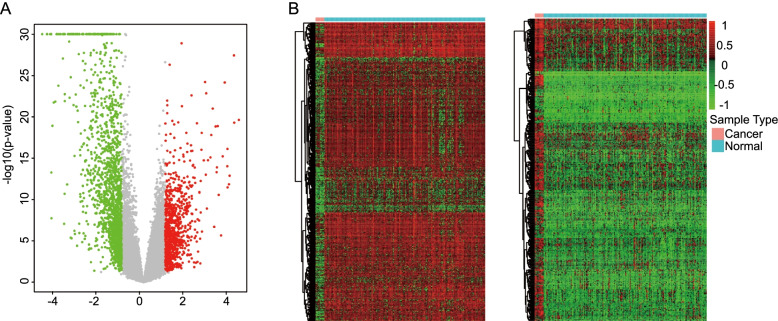
The DE-TFs was obtained based on the intersection of TFs database and DEGs between OC tissues and normal tissues. **A** The volcano plots of DEGs (*adj. P < 0.05* and *|logFC| > 1*). **B** The heatmaps showed a large number of TFs were either significantly up-regulated or down-regulated between OC samples and normal samples

We firstly collected the TFs in the JASPAR (612 TFs), TRANSFAC and CISBP (1639 TFs) and TRRUST (795 TFs) databases. After removing the duplicate TFs, the combined databases contained 1911 TFs. The data in GSE26712 was downloaded and the differentially expressed Genes (DEGs) were screened between OC tissues and normal tissues (*adj. P < 0.05* and *|logFC| > 0.05*). Genes with *|logFC| > 1* were presented as a volcano plot **(**Fig. 1A). Then the intersection between TFs database and DEGs was identified as the differentially expressed TFs (DE-TFs), with a total of 784 TFs. The up-regulated and down-regulated DE-TFs were presented as a heatmap **(**Fig. 1B). The results illustrated that a substantial number of TFs showed significant differences in expression levels between tumor tissues and normal tissues.

### Construction of TFs co-expression network

**Fig. 2:**
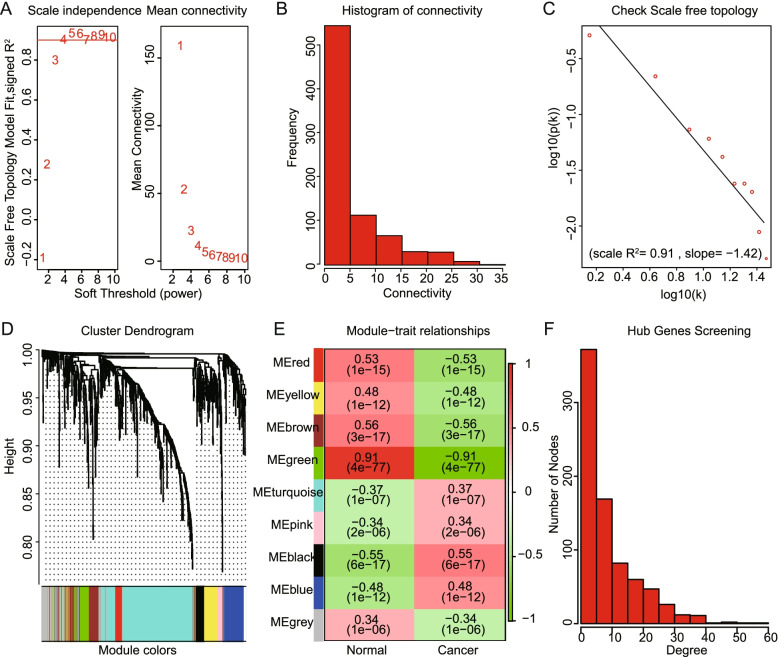
Identification of hub-TFs using WGCNA. **A** Screening optimal β-value to construct scale-free network. **B-C** Detection of network station. **D** Construction of WGCNA cluster modules. **E** Correlation analysis between cancers and normal samples in each module. **F** Identification of hub-TFs using cytoHubba add-in

To further obtain gene modules that strongly correlate with the cancers from the DE-TFs, the WGCNA was performed. The data meets the the scale-free condition and the optimal conditions was determined at β = 5. Then, we constructed the WGCNA network based on this condition to identify TFs data that is significantly correlated to the cancers from the DE-TFs. Next, 9 modules were constructed and 4 modules that have strong relevance were identified (green, brown, red and black (*cor > 0.5, P < 0.001)*). DE-TFs transcripts data in these 4 modules were further imported into Cytoscape for the recognition of hub-TFs. Finally, 70 DE-TFs with the highest gene node degree, defined as hub-TFs, were obtained and used for further analysis **(**Fig. 2**)**.

### GO functional enrichment analysis and KEGG pathway analysis

**Fig. 3:**
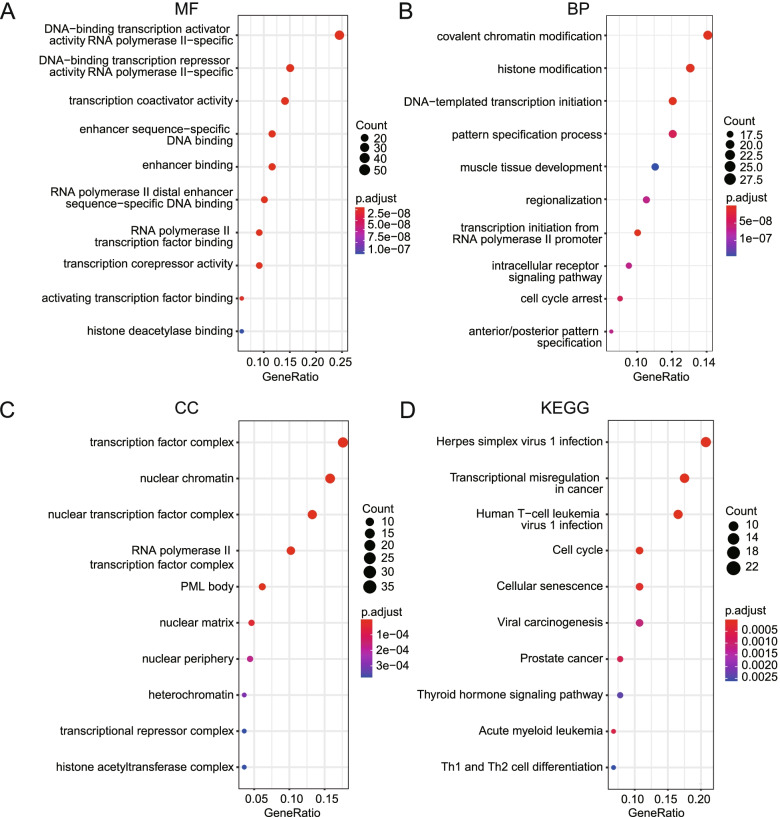
GO functional enrichment analysis and KEGG pathways analysis reveal that DE-TFs are involved in many cancer-related pathways. **A-C** top-10 significantly enriched GO terms and **D** top-10 significantly enriched KEGG pathways of DE-TFs. (MF, Molecular Function; BP, Biological Process; CC, Cellular component)

Functions and signal pathways of hub-TFs were conducted using GO functional enrichment analysis and KEGG pathway analysis **(**Fig. 3**)**. The result showed that the highest enriched GO terms in molecular function (MF), biological process (BP), and cellular component (CC) were DNA-binding transcription activator activity (*P < 0.001*), covalent chromatin modification (*P < 0.001*), and transcription factor complex (*P < 0.001*), respectively. The significantly enriched KEGG pathways included many cancer-related pathways, such as transcriptional mis-regulation in cancer (*P < 0.001*), cell cycle (*P < 0.001*), and viral carcinogenesis (*P < 0.01*). The results showed hub-TFs were mainly involved in various transcriptional process. Besides, hub-TFs were involved in pathways like cell cycle, cell senescence and histone modification. A large number of hub-TFs also participated in the regulation of many types of cancers, such as prostate cancer.

### Construction of TFs-based prognostic signature in patients with OC

**Fig. 4:**
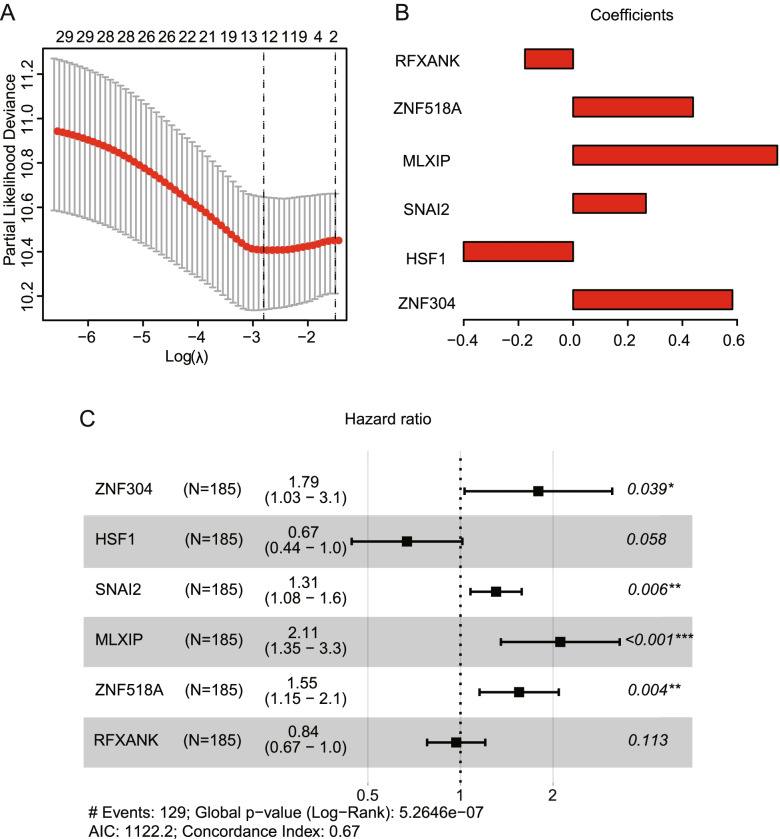
Identification of a 6 TFs prognostic signature using LASSO*-*Cox regression. **A** LASSO and **C** multivariate Cox regression was performed and **B** the coefficient of each TF in the prognostic signature was obtained

In order to construct a risk score assessment for predicting ovarian cancer prognosis based on above hub-TFs, we used the univariate Cox regression analysis in each hub-TF first, which identified 14 hub-TFs that were significantly correlated with clinical outcome of OC patients. Then, the model was further optimized through LASSO regression and multivariate Cox regression. Eventually, a prognostic signature was constructed containing 6 hub-TFs, including ZNF304, HSF1, SNAI2, MLXIP, ZNF518A and RFXANK **(**Fig. 4**)**. These 6 hub-TFs and their coefficients were showed in Table 2.

**Table 2:** The coefficient of hub-TFs containing in the prognostic prediction model

TFs	Coefficient
ZNF304	0.583
HSF1	−0.4
SNAI2	0.267
MLXIP	0.747
ZNF518A	0.439
RFXANK	−0.176

### The Prognostic ability of TFs-based signature

**Fig. 5:**
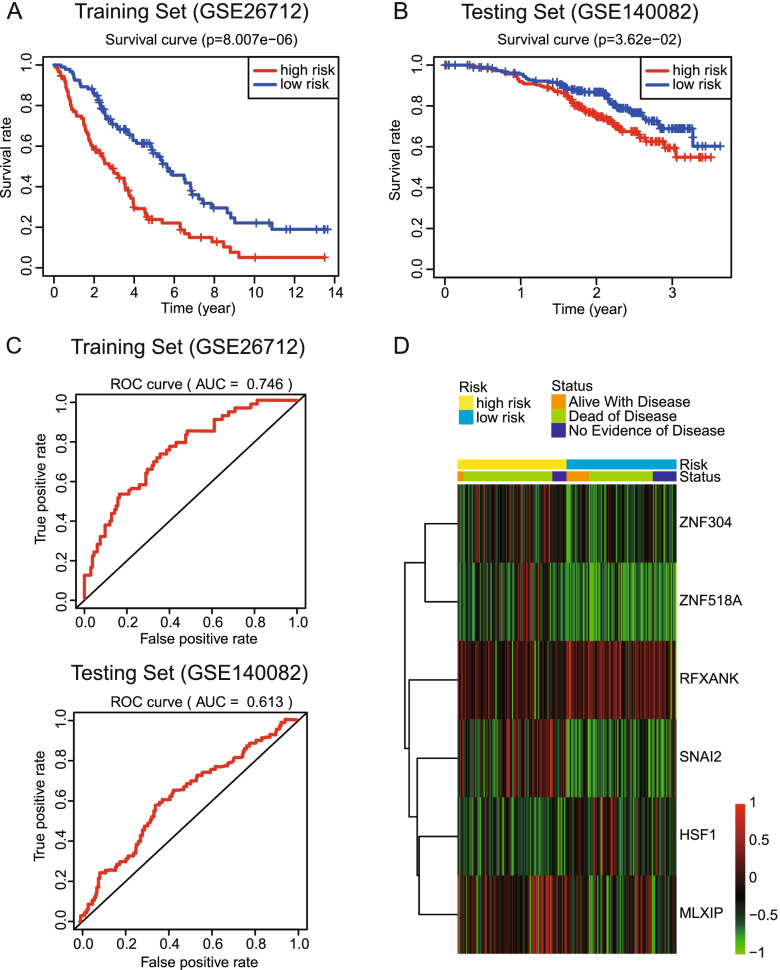
The evaluation of prognostic ability of TFs-based signature. **A**, **B** Kaplan-Merier survival curve of training set and validation set showed patients with higher risk have significantly worse clinical outcome. **C** Time-dependent ROC curves were conducted, and the TFs-based signature elevated the prognostic accuracy both in training and validation cohort. **D** Heatmap showed expression profile of 6 TFs in high-risk and low-risk groups

The predictive ability of the above prognostic signature in training set (GSE26712) and testing set (GSE140082) are shown in Fig. 5**.** The Kaplan-Merier survival curve revealed that high-risk patients had a significant worse survival in both training set (*P = 8.007E-6*) and testing set (*P = 0.0362*). In addition, the time-dependent ROC showed that the 2-year AUC of training cohort and validation cohort was 0.746 and 0.613, respectively. Moreover, the TFs expression profile of OC patients between high-risk and low-risk groups in training set was presented as a heatmap **(**Fig. 5D).

### Immune infiltration analysis between high-risk group and low-risk group

**Fig. 6:**
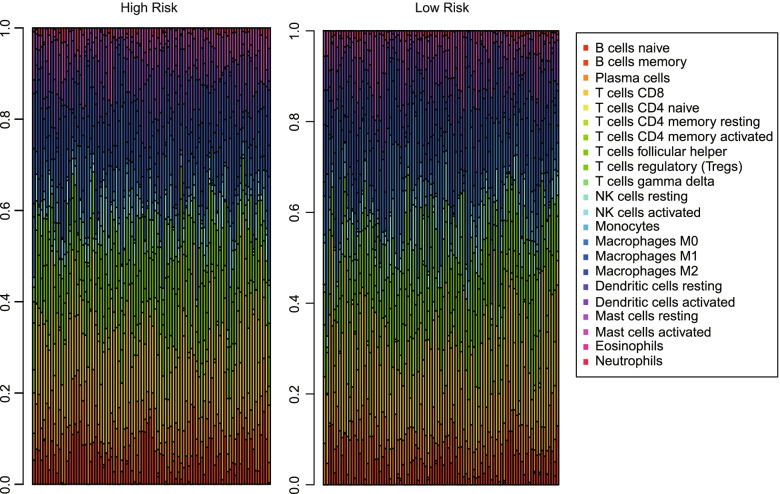
Immune infiltration analysis reveals association of TFs-based signature and 22 types tumor infiltrating immune cells between high-risk group and low-risk group

The association of TFs-based signature with tumor-infiltrating immune cells between high-risk group and low-risk group were further analyzed via CIBERSORT algorithm. An analysis of the profile of 22 types tumor infiltrating immune cells **(**Fig. 6**)** demonstrate that the abundance of B cells naïve, T cells CD4 memory resting, Macrophages M2 and Mast cells activated were significantly higher in high-risk group (*P < 0.05*). On the contrary, Macrophages M0 were significantly higher in low-risk group (*P < 0.001*) **(**Table 3), which suggest the expression of TFs might be correlated with tumor immune microenvironment and proportion of immune infiltrating cells might provide information for predicting the clinical outcome of OC patients.

**Table 3:** The relative abundance of 22 tumor immune cells between high-risk group and low-risk group

Cell types	*P*	Mean (high-risk group)	Mean (low-risk group)
B cells naive	0.028	0.055	0.04
B cells memory	0.699	0.01	0.011
Plasma cells	0.665	0.083	0.086
T cells CD8	0.747	0.18	0.174
T cells CD4 naive	NaN	0	0
T cells CD4 memory resting	0.004	0.04	0.027
T cells CD4 memory activated	0.564	< 0.001	< 0.001
T cells follicular helper	0.797	0.101	0.101
T cells regulatory (Tregs)	0.22	0.075	0.084
T cells gamma delta	0.929	0.005	0.008
NK cells resting	0.33	0.003	0.002
NK cells activated	0.202	0.042	0.05
Monocytes	0.276	0.017	0.015
Macrophages M0	< 0.001	0.059	0.106
Macrophages M1	0.82	0.054	0.055
Macrophages M2	0.021	0.092	0.074
Dendritic cells resting	0.055	0.068	0.055
Dendritic cells activated	0.958	0.051	0.052
Mast cells resting	0.511	0.007	0.009
Mast cells activated	0.021	0.051	0.042
Eosinophils	0.156	< 0.001	0
Neutrophils	0.903	0.006	0.006

### Construction of nomogram for predicting the prognosis of patients with OC

**Fig. 7:**
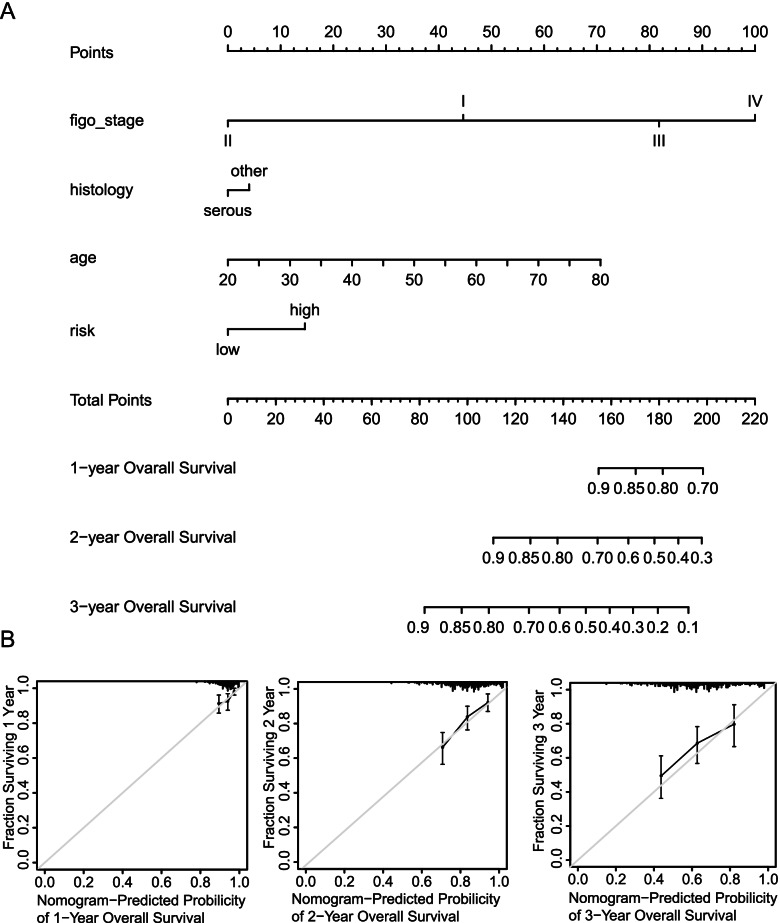
Nomogram for predicting overall survival of patients with OC according to TFs-based signature and clinical indicators. **A** Nomogram for predicting 1, 2- and 3-years overall survival of patients with OC. **B** Calibration curve for the nomogram predicting 1, 2- and 3-years overall survival with the ideal model

A nomogram was constructed, including stage, histology, age and TFs-based signature, to predict the overall survival probability of patients with OC in the training set **(**Fig. 7A)**.** According to the total-points-to-outcome nomogram, patients with higher number of total points were expected to have poorer 1, 2 and 3-year overall survival probability. The C-index of the nomogram was 0.664. In addition, calibration curves of the nomogram illustrated good predictive efficiencies for 1, 2, and 3-year overall survival **(**Fig. 7B).

### Construction of the TF-target gene regulatory network

**Fig. 8:**
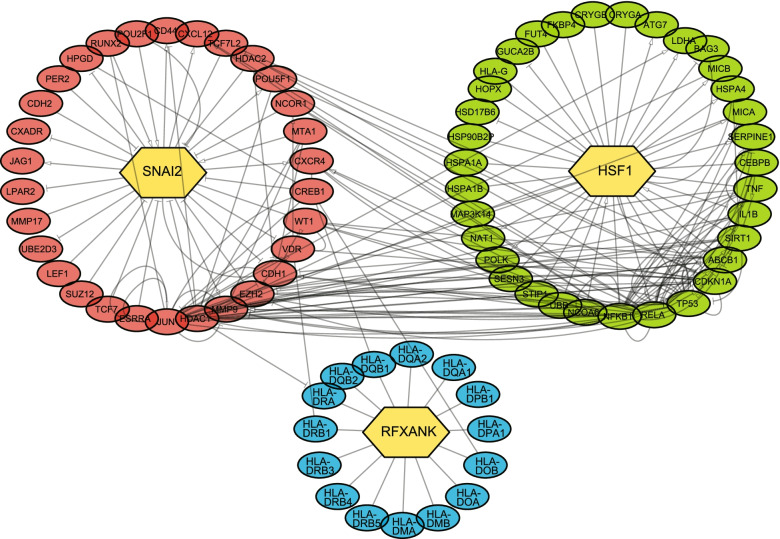
The construction of TF-target regulatory network of SNAI2, HSF1 and RFXANK and their possible mRNA targets

TFs in the prognostic signature may play pivotal roles in the OC pathogenesis. To understand the interaction of the TFs and their target genes in the OC, TF-target gene regulatory network was analyzed using TRRUST database. The regulatory network of SNAI2, HSF1 and RFXANK as well as their possible mRNA targets were visualized using Cytoscape. As shown in Fig. 8, the regulatory network contained 82 nodes and 220 edges, and they targeted different genes and participated in the regulation of biological functions relatively independently. Considering the high heterogeneity of OC, these suggested that the roles of the TFs in the prognostic signature may be versatile, which need to be further elucidated to provide more information for molecular mechanism and clinical research.

## Discussion

Ovarian cancer (OC) is one of the most common and lethal malignant tumors worldwide, with the second highest incidence rate and the highest mortality rate in all types of gynecological tumors. Despite the progress in imaging technologies, cytoreductive surgery and chemotherapy, the prognosis of OC remains poor, with less than half of 5-year survival rate. Early diagnosis of patients with OC is difficult owing to the absence of cancer-specific symptoms and effective screening tools. Patients with OC also suffered from relapse, high invasion and rapid drug resistance. Therefore, the improvement of individualized treatment is urgently needed. To this end, reliable, fast and accurate prognostic biomarkers of OC is critical for improving clinical stratification and determining personalize treatment options. Molecular markers, like genes, proteins, miRNAs and lncRNAs, which contribute to the pathogenesis of OC, are widely acknowledged as ideal candidates to predict the prognosis in OC patients and give clues to the underlying mechanism of OC. Due to the decrease of sequencing cost, the amount of data has rapidly increased in those years, which laid a solid foundation for the construction of a more accurate molecular-based prognostic model for predicting survival of patients with OC [13, 14]. It is also demonstrated that aberrant expression of TFs and dysregulation of its downstream targets is linked to the poor prognosis of many cancers including OC. Although TFs show potential prognostic value in OC, there have only been limited work investigating the roles of TFs in the clinical outcome and gene regulatory network in OC. Especially, the risk score model based on TFs in OC has been not reported so far. Therefore, we conduct a comprehensive bio-informatic analysis using data derived from GEO database in order to develop a reliable signature based on the TFs for predicting the prognosis of patients with OC.

Identification of TFs that play significant roles in tumorigenesis and progression of OC is the prerequisite for constructing a reliable and accurate risk score model. In order to minimize the potential missing of TFs, we identified DEGs in OC dataset on the one hand, and we acquired the combined TFs database on the other hand. Then, the first identification was completed by selecting the intersection between DEGs and TFs database and defined as DE-TFs. In order to obtain the TFs that are significantly associated with the OC among the DE-TFs, the WGCNA was used. The results showed that modules were successfully constructed, which strongly correlate with cancers. Then, the hub-TFs were selected from DE-TFs in cancer-related gene modules and the second identification was completed. We further screened the hub-TFs that are associated with the prognosis of OC using univariate Cox regression analysis. To construct the optimized risk score model, LASSO and stepwise multivariate Cox regression analysis were used to reduce the numbers of TFs. Finally, a TFs-based signature was generated after above three identifications. In previous studies, Zhao et al. screened 5 lncRNAs using WGCNA and Cox regression to construct a risk score system for prognosis assessment of OC [15]. Bao et al. developed a novel gene signature based on the DEGs that was enriched in G2/M checkpoint signaling pathway [16]. Compared to these studies, the initially included TFs were more comprehensive and the selection of TFs were more stringent in order to construct a more reliable and accurate prognostic model.

Our study established a TFs-based signature containing 6 hub-TFs, ZNF304, HSF1, SNAI2, MLXIP, ZNF518A and RFXANK. ZNF304 (Zinc Finger Protein 304) plays a role in gene silencing, including several tumor suppressor genes, and is overexpressed in several types of cancers. It has been reported that ZNF304 regulated β-1 integrin expression, promotes ovarian cancer cell survival and protects against anoikis in OC [17]. HSF1 (Heat Shock Factor Protein 1) is a transcription factor that regulates many heat shock proteins protecting cells from heat shock and other forms of chemical and physiological stress [18]. Targeting HSF1 revealed an antitumor effect and might be considered as a promising therapeutic strategy against OC [19, 20]. SNAI2 (Snail Family Transcriptional Repressor 2) is involved in epithelial-mesenchymal transitions and Wnt-mediated β-catenin signaling pathway. SNAI2 was found to induce EMT in ovarian cancer through suppressing miR-222-3p transcription and upregulating PDCD10 [21]. MLXIP (MLX Interacting Protein) forms a heterodimer with Max-like protein X (MLX) to activate transcription. MLXLP may play a role in ovarian cancer cell migration and was associated with prognosis [22]. ZNF518A (Zinc Finger Protein 518A) contains five zinc fingers and is likely a nuclear transcriptional regulator. It may mediate molecules that is crucial to both the development and maintenance of cell identity [23]. RFXANK (Regulatory Factor X Associated Ankyrin Containing Protein) binds to the MHC class II gene promoters and activates their transcription, which contributes to development and control of the immune system. RFXANK mutations were reported to be associated with stomach cancer and nasopharyngeal carcinoma [24, 25]. TFs included in the signature are all linked to cancers and have the potential to be novel diagnostic biomarkers and therapeutic targets. Furthermore, the TFs-target gene regulatory network of HSF1, SNAI2 and RFXANK was built to explore their connection with target genes. As expected, all three TFs target genes were highly linked to many oncogenes, tumor suppressors and cancer-related pathways. For instance, HSF1 participates in the regulation of TP53 and NF-κB. SNAI2 was also associated with CREB1 and MMP9. However, the regulatory network of these three TFs were relatively independent, which reveals the highly complex pathogenesis of OC.

Next, the TFs-based signature generated by our study was validated in both internal training dataset (GSE26712) and external dataset (GSE140082). Patients with OC are divided into high-risk group and low-risk group according to the level of risk score. Then, to validate the performance of the signature, Kaplan-Merier survival analysis and time-dependent ROC were conducted between the two groups. The results showed that high-risk patients had a significantly worse survival in both training set (*P < 0.001*) and testing set (*P = 0.0362*), with the 2-year AUC of 0.746 and 0.613, respectively. In the previous studies, several prognostic signatures for predicting survival in OC were built based on other molecular biomarkers, for instance, multi-gene signature including immune-related and energy metabolism-related gene signature, lncRNA signature and signaling pathway signature. Ding et al. constructed a signature containing nine genes related to tumor microenvironment with the 3-year AUC of 0.684 and 0.606 in the training cohort and testing cohort, respectively [26]. A multi-gene signature selected from hallmark gene set ‘HALLMARK G2M CHECKPOINT’ based on gene set enrichment analysis was validated by plotting time-dependent ROC curve and the 1-year AUC was 0.609 in GSE26712 [16]. Compared to above prognostic signatures, the TF-based signature showed a better prognostic accuracy in the training set but the AUC in the testing set needed to be further improved. Our validation results showed that TFs-based signature could serve as a promising candidate for predicting the survival of patients with OC and help the clinical decision-making.

The tumor immune microenvironment was identified to play a significant role in the progression and metastasis of OC, especially during the development of chemoresistance, which may present potential prognostic factors and therapeutic targets for ovarian cancer [27, 28]. The transcription and expression level of tumor tissues could represent the composition of various immune-related components. To explore the difference in tumor infiltration in OC between high-risk group and low-risk group divided by TFs-based signature, CIBERSORT algorithm was used to estimate 22 types tumor infiltrating immune cells composition from gene expression profiles in OC tumor tissues. Our study identified that the higher abundance of B cells naïve, T cells CD4 memory resting, Macrophages M2 and Mast cells were related to poor prognosis (*P < 0.05*). On the contrary, higher abundance of Macrophages M0 were related to favorable prognosis (*P < 0.05*). Previous studies showed lymphocytes infiltration was associated with prognosis of patients with OC [29]. It was found that M2-type macrophages infiltrating metastatic sites could limit immune responses against OC [30]. M2-type macrophages also enhanced the proliferation, invasion and migration, and inhibited apoptosis of OC cancer in vitro [31]. Limiting the tumor-promoting activity of M2-type macrophages is a promising OC therapy by targeting tumor microenvironment [32]. Our finding revealed the potential of tumor infiltrating immune cells like M2-type macrophages for predicting the survival of patient with OC and the hub-TFs may play a role in tumor immune microenvironment in OC.

Limitations in our study including the following: 1) although Kaplan-Merier survival curve illustrated a significantly poorer outcome in patients with OC in the high-risk group according to the TFs-based signature, it is necessary to improve the performance of AUC in the testing datasets, like 1-year and 5-year. Therefore, our TFs-based signature needed to be further validated and updated to elevate the prognostic ability. The TFs-based signature could also be improved when there are more appropriate datasets in the future. 2) despite the construction of the TFs-target gene regulatory network, the molecular mechanism behind the network need to be further elucidated in in vitro and in vivo experiments. Moreover, in order to further help the clinical stratification and personalize treatment options of patients with OC, it is reasonable to combine our TFs-based signature with more factors, such as lncRNAs, and clinicopathological parameters to continue developing the prognostic risk score model of OC.

## Conclusion

Our study established the first TFs-based signature that might be effective in the prediction of prognosis in patients with OC. The signature contains 6 hub-TFs, which are identified mainly using differential expression analysis, WGCNA and Cox-LASSO regression analysis. The TFs-based signature was validated. A nomogram based on the signature was constructed and demonstrated good performance in predicting survival of patients with OC. Furthermore, immune infiltrating analysis showed that TFs included in the signature may play a role in the tumor immune microenvironment. In addition, the TFs-target gene regulatory network suggested that these TFs participate in many cancer-related pathways. Our study could provide helpful information for the clinical stratification and personalized treatment options in patients with OC. It also gives insights into the potential roles of TFs in the molecular mechanism of OC.

## Supplementary Information

**Additional file 1: Table S1** Characteristics of patients with ovarian cancer in GSE140082

## Data Availability

The datasets generated and analyzed during the current study are available in the [GEO database] repository, [https://www.ncbi.nlm.nih.gov/gds/].

## Abbreviations

OC: Ovarian cancer
TFs: Transcription factors
DE-TFs: Differentially expressed TFs
WGCNA: Weighted Correlation Network Analysis
GO: Gene Ontology
KEGG: Kyoto Encyclopedia of Genes and Genomes
LASSO: Least absolute shrinkage and selection operator
ROC: Receiver operating characteristic

## References

[1] Bray F, Ferlay J, Soerjomataram I, Siegel RL, Torre LA, Jemal A (2018). Global cancer statistics 2018: GLOBOCAN estimates of incidence and mortality worldwide for 36 cancers in 185 countries. CA Cancer J Clin.

[2] Webb PM, Jordan SJ (2017). Epidemiology of epithelial ovarian cancer. Best Pract Res Clin Obstet Gynaecol.

[3] Kroeger PT, Drapkin R (2017). Pathogenesis and heterogeneity of ovarian cancer. Curr Opin Obstet Gynecol.

[4] Belluti S, Rigillo G, Imbriano C (2020). Transcription Factors in Cancer: When Alternative Splicing Determines Opposite Cell Fates. Cells..

[5] Vishnoi K, Viswakarma N, Rana A, Rana B (2020). Transcription Factors in Cancer Development and Therapy. Cancers (Basel)..

[6] Wan Y, Liu H, Zhang M, Huang Z, Zhou H, Zhu Y, Tao Y, Xie N, Liu X, Hou J, Wang C (2020). Prognostic value of epithelial-mesenchymal transition-inducing transcription factors in head and neck squamous cell carcinoma: A meta-analysis. Head Neck.

[7] Sun CC, Li SJ, Hu W, Zhang J, Zhou Q, Liu C, Li LL, Songyang YY, Zhang F, Chen ZL (2019). Comprehensive Analysis of the Expression and Prognosis for E2Fs in Human Breast Cancer. Mol Ther.

[8] Wang JY, Lu AQ, Chen LJ (2019). LncRNAs in ovarian cancer. Clin Chim Acta.

[9] Deb B, Uddin A, Chakraborty S (2018). miRNAs and ovarian cancer: An overview. J Cell Physiol.

[10] Nameki R, Chang H, Reddy J, Corona RI, Lawrenson K. Transcription factors in epithelial ovarian cancer: histotype-specific drivers and novel therapeutic targets. Pharmacol Ther. 2021;220:107722. 10.1016/j.pharmthera.2020.107722 33137377

[11] Guo Q, Wang J, Gao Y, Li X, Hao Y, Ning S, Wang P (2020). Dynamic TF-lncRNA Regulatory Networks Revealed Prognostic Signatures in the Development of Ovarian Cancer. Front Bioeng Biotechnol.

[12] Guo Q, He Y, Sun L, Kong C, Cheng Y, Wang P, Zhang G (2019). Identification of potential prognostic TF-associated lncRNAs for predicting survival in ovarian cancer. J Cell Mol Med.

[13] Kim SI, Song M, Hwangbo S, Lee S, Cho U, Kim JH, Lee M, Kim HS, Chung HH, Suh DS (2019). Development of Web-Based Nomograms to Predict Treatment Response and Prognosis of Epithelial Ovarian Cancer. Cancer Res Treat.

[14] Bookman MA (2019). Can we predict who lives long with ovarian cancer?. Cancer.

[15] Zhao Q, Fan C (2019). A novel risk score system for assessment of ovarian cancer based on co-expression network analysis and expression level of five lncRNAs. BMC Med Genet.

[16] Bao M, Zhang L, Hu Y (2020). Novel gene signatures for prognosis prediction in ovarian cancer. J Cell Mol Med..

[17] Aslan B, Monroig P, Hsu MC, Pena GA, Rodriguez-Aguayo C, Gonzalez-Villasana V, Rupaimoole R, Nagaraja AS, Mangala S, Han HD (2015). The ZNF304-integrin axis protects against anoikis in cancer. Nat Commun.

[18] Rabindran SK, Giorgi G, Clos J, Wu C (1991). Molecular cloning and expression of a human heat shock factor, HSF1. Proc Natl Acad Sci U S A.

[19] Chen YF, Wang SY, Yang YH, Zheng J, Liu T, Wang L (2017). Targeting HSF1 leads to an antitumor effect in human epithelial ovarian cancer. Int J Mol Med.

[20] Wilson AL, Moffitt LR, Duffield N, Rainczuk A, Jobling TW, Plebanski M, Stephens AN (2018). Autoantibodies against HSF1 and CCDC155 as Biomarkers of Early-Stage, High-Grade Serous Ovarian Cancer. Cancer Epidemiol Biomark Prev.

[21] Fan L, Lei H, Zhang S, Peng Y, Fu C, Shu G, Yin G (2020). Non-canonical signaling pathway of SNAI2 induces EMT in ovarian cancer cells by suppressing miR-222-3p transcription and upregulating PDCD10. Theranostics.

[22] Meunier L, Puiffe ML, Le Page C, Filali-Mouhim A, Chevrette M, Tonin PN, Provencher DM, Mes-Masson AM (2010). Effect of ovarian cancer ascites on cell migration and gene expression in an epithelial ovarian cancer in vitro model. Transl Oncol.

[23] Maier VK, Feeney CM, Taylor JE, Creech AL, Qiao JW, Szanto A, Das PP, Chevrier N, Cifuentes-Rojas C, Orkin SH (2015). Functional Proteomic Analysis of Repressive Histone Methyltransferase Complexes Reveals ZNF518B as a G9A Regulator. Mol Cell Proteomics.

[24] Yavorski JM, Blanck G (2017). MHC class II associated stomach cancer mutations correlate with lack of subsequent tumor development. Mol Clin Oncol.

[25] Zhou P, Liu S, Ji NN, Zhang S, Wang P, Lin B, Yang P, Lin XT, Cai YZ, Wang ZM (2020). Association between variant alleles of major histocompatibility complex class II regulatory genes and nasopharyngeal carcinoma susceptibility. Eur J Cancer Prev.

[26] Ding Q, Dong S, Wang R, Zhang K, Wang H, Zhou X, Wang J, Wong K, Long Y, Zhu S (2020). A nine-gene signature related to tumor microenvironment predicts overall survival with ovarian cancer. Aging (Albany NY).

[27] Zhang B, Chen F, Xu Q, Han L, Xu J, Gao L, Sun X, Li Y, Li Y, Qian M, Sun Y (2018). Revisiting ovarian cancer microenvironment: a friend or a foe?. Protein Cell.

[28] Horowitz M, Esakov E, Rose P, Reizes O (2020). Signaling within the epithelial ovarian cancer tumor microenvironment: the challenge of tumor heterogeneity. Ann Transl Med.

[29] Santoiemma PP, Powell DJ (2015). Tumor infiltrating lymphocytes in ovarian cancer. Cancer Biol Ther.

[30] Hensler M, Kasikova L, Fiser K, Rakova J, Skapa P, Laco J, et al. M2-like macrophages dictate clinically relevant immunosuppression in metastatic ovarian cancer. J Immunother Cancer. 2020;8(2):e000979. 10.1136/jitc-2020-000979 PMC7443306 32819974

[31] Yi J, Lin Y, Yicong W, Chengyan L, Shulin Z, Wenjun C (2020). Effect of macrophages on biological function of ovarian cancer cells in tumor microenvironment in vitro. Arch Gynecol Obstet.

[32] Yang Y, Yang Y, Yang J, Zhao X, Wei X (2020). Tumor Microenvironment in Ovarian Cancer: Function and Therapeutic Strategy. Front Cell Dev Biol.

