# Supplementary material for: Construction and validation of a transcription factors-based prognostic signature for ovarian cancer
Source: J Ovarian Res. 2022 Feb 28;15:29. doi: 10.1186/s13048-021-00938-2 (PMC8886838; doi:10.1186/s13048-021-00938-2)
Supplement: Supplementary file 1 — Additional file 1: Table S1 Characteristics of patients with ovarian cancer in GSE140082 [file 13048_2021_938_MOESM1_ESM.docx]

Table S1 Characteristics of patients with ovarian cancer in GSE140082

| Patient characteristics | Sample Count | Portion |
| --- | --- | --- |
| Age |  |  |
| 21-30 | 7 | 0.0184 |
| 31-40 | 17 | 0.0447 |
| 41-50 | 79 | 0.2079 |
| 51-60 | 106 | 0.2789 |
| 61-70 | 137 | 0.3605 |
| 71-80 | 34 | 0.0895 |
| Histology |  |  |
| serous | 277 | 0.7289 |
| other | 103 | 0.2711 |
| FIGO_stage |  |  |
| I | 20 | 0.0526 |
| II | 31 | 0.0816 |
| III | 266 | 0.7000 |
| IV | 63 | 0.1658 |
| Molecular subtype^a^ |  |  |
| Mesenchymal | 25 | 0.0658 |
| Immunoreactive | 124 | 0.3263 |
| Differentiated | 86 | 0.2263 |
| Proliferative | 97 | 0.2553 |

^a^phenotypically distinct expression-based high grade serous tubo-ovarian carcinoma
